# A 3D-QSAR Study on the Antitrypanosomal and Cytotoxic Activities of Steroid Alkaloids by Comparative Molecular Field Analysis

**DOI:** 10.3390/molecules23051113

**Published:** 2018-05-08

**Authors:** Charles Okeke Nnadi, Julia Barbara Althaus, Ngozi Justina Nwodo, Thomas Jürgen Schmidt

**Affiliations:** 1 Institute of Pharmaceutical Biology and Phytochemistry (IPBP), University of Münster, PharmaCampus Corrensstraße 48, D-48149 Münster, Germany; charles.nnadi@unn.edu.ng (C.O.N.); julia.althaus@uni-muenster.de (J.B.A.); 2 Department of Pharmaceutical and Medicinal Chemistry, Faculty of Pharmaceutical Sciences, University of Nigeria, Nsukka, Enugu State 410001, Nigeria; ngozi.nwodo@unn.edu.ng

**Keywords:** aminosteroid, aminocycloartane, alkaloid, 3D-QSAR, CoMFA, antitrypanosomal activity, cytotoxicity

## Abstract

As part of our research for new leads against human African trypanosomiasis (HAT), we report on a 3D-QSAR study for antitrypanosomal activity and cytotoxicity of aminosteroid-type alkaloids recently isolated from the African medicinal plant *Holarrhena africana* A. DC. (Apocynaceae), some of which are strong trypanocides against *Trypanosoma brucei rhodesiense* (*Tbr*), with low toxicity against mammalian cells. Fully optimized 3D molecular models of seventeen congeneric *Holarrhena* alkaloids were subjected to a comparative molecular field analysis (CoMFA). CoMFA models were obtained for both, the anti-*Tbr* and cytotoxic activity data. Model performance was assessed in terms of statistical characteristics (*R*^2^, *Q*^2^, and *P*^2^ for partial least squares (PLS) regression, internal cross-validation (leave-one-out), and external predictions (test set), respectively, as well as the corresponding standard deviation error in prediction (SDEP) and *F*-values). With *R*^2^ = 0.99, *Q*^2^ = 0.83 and *P*^2^ = 0.79 for anti-*Tbr* activity and *R*^2^ = 0.94, *Q*^2^ = 0.64, *P*^2^ = 0.59 for cytotoxicity against L6 rat skeletal myoblasts, both models were of good internal and external predictive power. The regression coefficients of the models representing the most prominent steric and electrostatic effects on anti-*Tbr* and for L6 cytotoxic activity were translated into contour maps and analyzed visually, allowing suggestions for possible modification of the aminosteroids to further increase the antitrypanosomal potency and selectivity. Very interestingly, the 3D-QSAR model established with the *Holarrhena* alkaloids also applied to the antitrypanosomal activity of two aminocycloartane-type compounds recently isolated by our group from *Buxus sempervirens* L. (Buxaceae), which indicates that these structurally similar natural products share a common structure–activity relationship (SAR) and, possibly, mechanism of action with the *Holarrhena* steroids. This 3D-QSAR study has thus resulted in plausible structural explanations of the antitrypanosomal activity and selectivity of aminosteroid- and aminocycloartane-type alkaloids as an interesting new class of trypanocides and may represent a starting point for lead optimization.

## 1. Introduction

Understanding the structure–activity relationships (SARs) of bioactive molecules is a crucial prerequisite for lead optimization and further drug development. We recently reported on aminosteroid-type alkaloids from West African *Holarrhena africana* A. DC. (a synonym of *H. floribunda* (G. Don) T. Durand and Schinz) as a novel class of antitrypanosomal compounds with considerable activity against *Trypanosoma brucei rhodesiense* (*Tbr*) and low cytotoxicity which may represent a promising starting point towards new drugs against human African trypanosomiasis (HAT) [1]. In our first report, we also described some elementary structure–activity relationships, already perceived when comparing the structures and biological potency of the compounds. The mechanism of action and biological target for the antitrypanosomal activities of these compounds are still unknown and quantitative structure–activity relationships (QSAR) studies may represent the first step towards a better understanding of their promising bioactivity. QSAR studies play an important role in ligand-based drug discovery, design, and further development [2,3]. Comparative molecular field analysis (CoMFA) represents the earliest and most widely applied three dimensional (3D)-QSAR method [4]. It employs statistical techniques to correlate biological endpoints with molecular features in terms of calculated interaction energies with virtual probes and interactive graphics can be used subsequently to interpret the results. Notably, the CoMFA method, originally developed with a set of steroids [5], has frequently been applied to numerous steroids and various biological endpoints [6,7,8,9,10,11,12,13], so that it appeared straightforward to apply it to the present set of alkaloids with pregnane and pregnene scaffolds. The compounds under study can roughly be grouped into (a) androstane and androst-5-ene derivatives with 3-amino- and 17β-acetyl substituents, (b) pregn-5-ene derivatives with a 3-amino substituent and an additional amino group connecting C-20 and C-18 forming a pyrrolidine ring and (c) pregnene derivatives with a C-3- or C-7-oxo substituent and a C-18/C-20 pyrrolidine or pyrroline ring similar to (b). The comparatively low flexibility within the steroid nucleus of these molecules makes their alignment, which is a most crucial step in 3D-QSAR, particularly in CoMFA, quite straightforward. The structural congenericity of the compounds in our data set furthermore makes it likely that they act by a common single target mediated mechanism. The corresponding endpoint data originate from a congruent series of determinations in the same laboratory and cover a considerable range of potency (2.5 log units in case of the antitrypanosomal activity). At the same time, the variations in substitution of the steroid core and a reasonable number of molecules in our set make it appear feasible to capture the most relevant structure–activity associations for both the antitrypanosomal as well as the cytotoxic activity using a 3D-QSAR method such as CoMFA, which could, in future studies, be used to attempt predictions for (semi-) synthetic modifications to further optimize the activity profile.

## 2. Results and Discussion

### 2.1. Modeling and Alignment of Molecular Structures 

The structures and activity data of the *Holarrhena* alkaloids under study against *Tbr* and L6 rat skeletal myoblasts are presented in Table 1. The data were divided into a training set to generate the CoMFA models and a test set for subsequent external validation.

**Figure 1:**
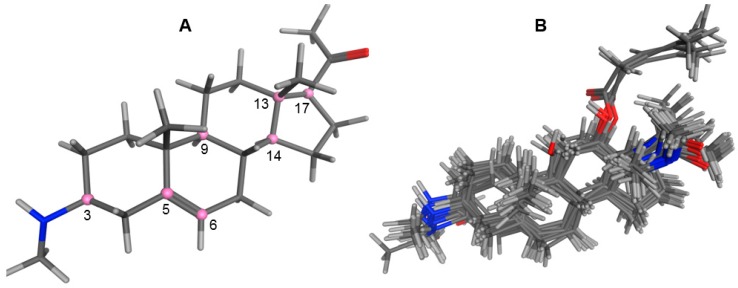
(**A**) 3D model of **3** used as alignment template. Selected atoms (marked) were used as matching points for the superposition; (**B**) Superposed 3D structures of all steroid alkaloids under study.

An optimized molecular model of each compound was generated using the Molecular Operations Environment (MOE [14]). The lowest energy conformer of the most active molecule (**3**, Figure 1A) served as the template scaffold for atom-by-atom alignment of all steroids. The atoms of the steroid nucleus marked in Figure 1A were used as alignment points for the compound superposition, which is shown in Figure 1B.

### 2.2. CoMFA Modelling

The molecular structures were exported to the software Open3DQSAR [15]. After calculation of molecular interaction fields (MIFs) with a steric and an electrostatic probe and various data pre-treatment steps, partial least squares (PLS) regression with up to five latent variables (PLS components) was performed to model linear relationships between the differences in the molecules’ MIF energies with the changes in the trypanocidal (Tbr) and cytotoxic (L6) activities. The resulting models were cross-validated using the leave-one-out (LOO) method and the optimum number of components in the models for each activity was selected based on coefficients of determination (Q^2^) of the cross validation predictions. The predictive ability of the models was furthermore assessed by activity predictions for the test sets of compounds excluded during CoMFA model generation. The general statistical parameters of the two resulting models are summarized in Table 2; More details on each model and the predicted pIC_50_ values of all compounds in both models are reported as Supplementary Materials (Tables S1–S3).

#### 2.2.1. PLS and Model Statistics for Anti-Tbr Activities of Steroid Alkaloids

**Figure 2:**
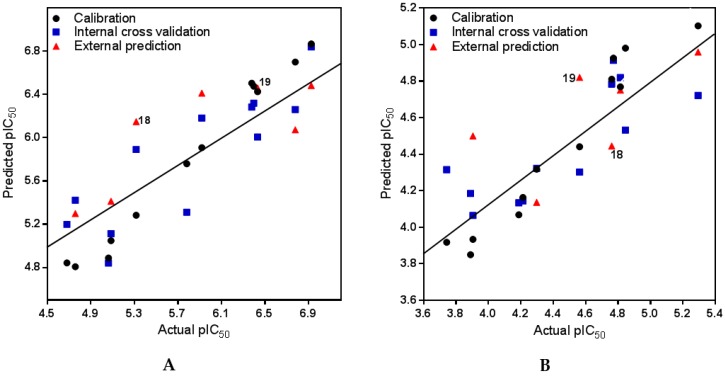
Scatter plots of predicted versus actual activity of steroid alkaloids against *Tbr* (**A**) and L6 rat skeletal myoblasts (**B**). Data result from the partial least squares (PLS) models with a 3 (**A**) and a 2 (**B**) components and represent non-cross validated (black circles), internal predictions by leave-one-out (LOO) cross validation (blue squares) and test set predictions (red triangles). The positions of the *Buxus* alkaloids **18** and **19** (see Section 2.4) are marked. The trendlines are for the non-cross validated data. For numerical values of all compounds see Table S3, Supplementary Materials.

The best model developed for trypanocidal (*Tbr*) activity comprised an optimum number of three latent variables (PLS components, PCs) and confirmed a strong correlation between the variations in the MIFs and the in vitro activities of the steroid alkaloids. The PLS regression yielded coefficients of determination, *R*^2^ = 0.99, *Q*^2^ = 0.83, and *P*^2^ of 0.79 for the model calibration, the leave-one-out internal cross validation, and for the predictions of the test set compounds’ activities, respectively. The model statistics are summarized in Table 2 and a plot of predicted versus measured pIC_50_ values is presented in Figure 2A.

#### 2.2.2. PLS and Model Statistics for L6 Cytotoxic Activities of Steroid Alkaloids

The CoMFA model for cytotoxic activities of steroid alkaloids on L6 rat skeletal myoblasts also yielded a strong PLS correlation with *R*^2^ = 0.94, *Q*^2^ = 0.64. For this model only two PLS components were required, since no further significant increase of *Q*^2^ was observed in a three-component model. The predictive ability of this two-component model gave a coefficient of determination *P*^2^ of 0.59. The somewhat lower statistical quality of this regression model in comparison with the one for anti-*Tbr* activity may be explained by the more narrow range of biological data (≈1.9 log units). For model statistics see Table 2 and for a plot of predicted versus measured activity data see Figure 2B.

Most importantly, the high *F*-values and SDEP <*P*^2^ for both models indicate that the possibility of chance correlation in the models is small. More so, the closeness of the coefficients of determination for internal (*Q*^2^) validation and the external (*P*^2^) predictions confirms the robustness of the models.

### 2.3. Analysis of the CoMFA Contour Maps

**Figure 3:**
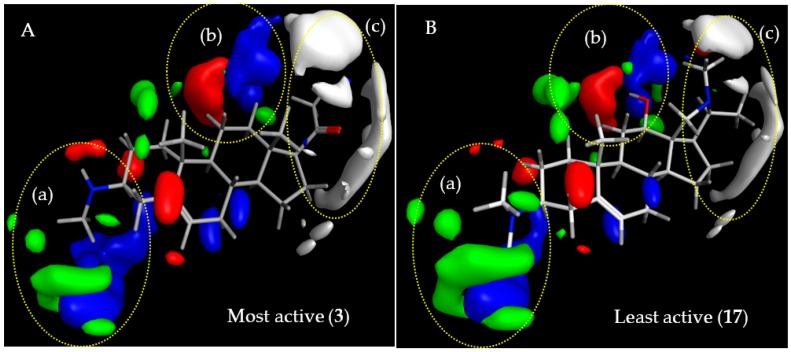
Steric and electrostatic contour maps representing the comparative molecular field analysis (CoMFA) model for anti-*Tbr* activity. Compounds shown are the strongest (**A**; **3**) and weakest (**B**; **17**) trypanocides in the series. Green and white regions, respectively, indicate areas where steric interactions increase and decrease activity. Blue and red regions denote enhancing and detrimental electrostatic effects with the positively charged probe, respectively.

**Figure 4:**
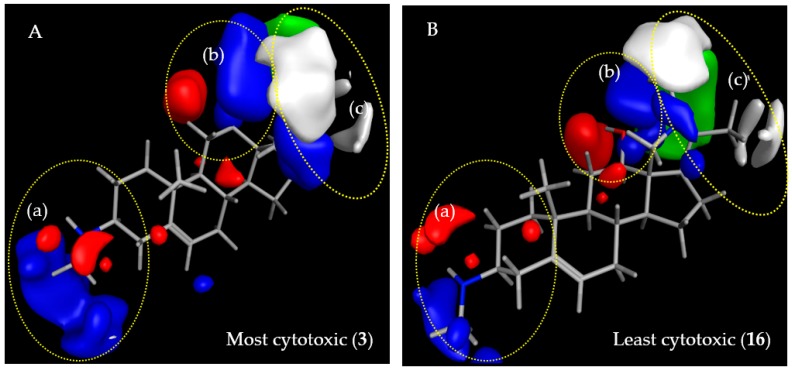
Steric and electrostatic contour maps representing the CoMFA model for cytotoxic activity on L6 rat skeletal myoblasts. Compounds shown are the strongest (**A**; **3**) and weakest (**B**; **16**) cytotoxins in the series. Green and white regions, respectively, indicate areas where steric interactions increase and decrease activity. Blue and red regions denote enhancing and detrimental electrostatic effects with the positively charged probe, respectively.

The regression coefficients of the final CoMFA models for the steroid alkaloids were translated into contour maps for both steric and electrostatic effects on antitrypanosomal (*Tbr*) activity and cytotoxic activity on L6 cells (see Figure 3 and Figure 4, respectively). The contours reflect properties of a hypothetical common receptor binding site where variations of steric and electrostatic features of the molecules affect most significantly the antitrypanosomal and cytotoxic activities of the compounds and give some general insight into the nature of the putative common receptor-ligand binding region.

#### 2.3.1. CoMFA Model for Antitrypanosomal Activity of Steroid Alkaloids

Contributions by steric effects are observed mainly in the vicinities of the C-3 amino group (region (a) in Figure 3, where a steric interaction tends to increase activity) and around the amino group of the pyrroline/pyrrolidine rings and the C-17β-acetyl or C-20 methyl groups (region (c) in Figure 3) where steric interaction tends to decrease activity. Region (a) accounts for interactions of methyl groups bound to the amino substituents in position C-3 where monomethylation significantly enhances the activity (compare **3** with **1** and **5**). On the other hand, methyl substituents on the pyrroline/pyrrolidine ring of the weaker trypanocides (**17** shown in Figure 3B) protrude into the region (c) of sterically unfavorable interaction and obviously reduce this activity.

Prominent electrostatic effects are observed around the C-3 amino position (Figure 3, blue area in region (a)) and in the vicinity of the C-11/C-12 position of the steroid skeleton (Figure 3, red and blue areas in region (b); The N-atom of the C-18-N-C-20 bridge may also interact with the latter but the distance is relatively large and so that this is obviously of less importance since the activity is not strongly influenced by this structure element)). Electrostatic interaction with a positive charge on the putative receptor is favorable for antitrypanosomal activity mainly in the vicinity of the amino groups at C-3 while it can be either detrimental or favorable in the C-11/C-12 and C-18-N-C-20 area (red and blue areas in region (b), respectively).

#### 2.3.2. CoMFA Model for L6 Cytotoxic Activity of Steroid Alkaloids

The steric contour map of the L6 cytotoxic activities (Figure 4) is completely governed by interactions in the C-17 region (region (c) in Figure 4). The additional pyrroline/pyrrolidine rings of **7**–**17** protrude with their methyl substituents to the white (sterically unfavorable) region while the C-17β-acetyl groups of **1**–**6** only protrude into the green (sterically favorable) contour in this region suggesting that this substituent enhances cytotoxicity. Similarly, the 12-*O*-(4′-methyl-3′-pentenoyl) ester chain of **14** and **15** protrudes to the green region with the ∆^3′,4′^-double bond partially touching the sterically favorable region (not shown).

The electrostatic contour maps for the L6 cytotoxic activities of the steroid alkaloids resemble those in the model for anti-*Tbr* activity with an enhancing influence of region (a) near the C-3 amino group. The amino groups at position C-3 interact with the blue contour region so that they have a similar effect on cytotoxicity as on antitrypanosomal activity while the amino groups of the pyrroline/pyrrolidine rings are somewhat more remote from the respective blue contour in the region extending in regions (b) and (c) where interactions with electropositive charge would favor the cytotoxic activity. The hydroxy group of the C-12 alcohols **16**/**17** is located between a red (unfavorable) and a blue (favorable) region while the ester oxygens of their counterparts **14**/**15** protrude more into the blue region (not shown).

#### 2.3.3. Comparison of CoMFA Models and Considerations on Antitrypanosomal Selectivity of Steroid Alkaloids

As becomes clear from the previous sections, the CoMFA models for the two activities under study are relatively similar indicating that the putative biological receptor sites are related but differ enough to warrant considerable selectivity in some compounds. As already pointed out, in region (a), interaction of the C-3 amino group with a positive charge (blue region) on the receptor site is favored for both activities, which confirms our earlier postulation [1] that such substituents at the C-3 position are required for strong antitrypanosomal activity of steroid alkaloids against *Tbr* but also confer cytotoxicity. The green contour in region (a), observed only in the *Tbr* model, Figure 3, indicates that there might be a favorable steric interaction with the methyl groups of C-3-methylamino groups but the higher activity of such methylamines could equally be caused by their increased basicity, which appears even more likely since activity increases in the order –NH_2_ < –N(CH_3_)_2_ < –NHCH_3_ and not with the number of methyl groups. The lower anti-*Tbr* activity and cytotoxicity of the acetamide **2** compared with **1** and **3** must be attributed to a much weaker electrostatic interaction of the non-basic amide nitrogen in this region. Along the same lines, the necessity for electrostatic interactions with a basic group in region (a) for high anti-*Tbr* activity is also reflected in the comparatively low activities of **7**–**10**, none of which bears an amino group in this region. Compounds **7**–**9** are 3-oxosteroids and **10** is a 7-oxosteroid with only a hydrogen at C-3. Thus, the influences on activity of interactions in region (a) on anti-*Tbr* and cytotoxic activity appear to parallel each other suggesting that this position could not easily be further optimized to increase the selectivity towards *Tbr*.

While interactions in region (b) of both models are rather similar and may thus not yield any good points of attack for selectivity, the two models indicate that region (c) may represent a viable target in this respect. Interestingly, cytotoxic activity is most prominently influenced by both steric and electrostatic interactions in region (c) (Figure 4) comprising the pyrroline/pyrrolidine ring of **11**–**17** and the C-17β-acetyl substituent of **1**–**6**. The higher cytotoxic activity observed in compounds **1**–**6** in comparison with the pentacyclic diamines **11**–**17**, according to the model, may be explained by a significant steric contribution of the C-17β-acetyl group whose methyl group points towards a green area of favorable steric interaction in the L6 model, alongside a favorable electrostatic interaction of the keto oxygen with a blue electropositive region, which is larger than in the *Tbr* model and extends in both regions (b) and (c). An electrostatic interaction within this region should also be possible for the amino nitrogen in the pyrroline/pyrrolidine ring connecting C-18 and C-20 of **11**–**17**, which might hence tend to increase the cytotoxic activity of the compounds but this is apparently outmatched by the stronger steric effect of the methyl substituents at C-20 and the nitrogen atom, which point into an extended white area of unfavorable Van der Waals interaction. Nevertheless, it could be speculated that replacement of the nitrogen between C-18 and C-20 by, e.g., a methylene group, could further decrease the cytotoxicity because the mentioned electrostatic effect in this case would not be possible. A white contour area of detrimental steric interaction is also observed in the anti-*Tbr* model but is apparently of less influence. The pyrrolidine/pyrroline structure element in region (c) and, in particular, the methyl groups attached to it thus appear to reduce the cytotoxicity to a greater extent than antitrypanosomal activity and thereby to increase the selectivity of the alkaloids bearing this structural feature. While the C-17β-acetyl substituent of the pregnane/pregnene derivatives (**1**–**6**) as well as the additional ring in (**7**–**17**) is obviously well accommodated, this region of detrimental steric effect in the anti-*Tbr* model appears to be more sensitive to the differences in methylation at the C-18/20 nitrogen bridge. This is in line with the higher activity of **11** compared with **13**. These two compounds differ by the presence and absence of a methyl substituent on the pyrrolidine nitrogen in **13** and **11**, respectively. The unmethylated **11** yielded an IC_50_ value against *Tbr* of 0.17 µM while that of the methylated pyrollidine **13** was 0.42 µM indicating that this *N*-methyl group is responsible for an unfavorable steric interaction in region (c). Taking into account that compounds with a monomethylamino group at C-3 (region (a)) show a higher anti-*Tbr* activity than the respective dimethylamines, it may hence be hypothesized, that a derivative of the C-3-monomethylamine **12** without the *N*-methylation at the pyrrolidine nitrogen would be even more active since **12** already as such has an IC_50_ of only 0.166 µM. At the same time, the increase in cytotoxicity upon removal of this methyl group is not significant so that a further increased selectivity can be expected for *N*^18^-demethyl-**12**.

### 2.4. Application of the CoMFA Model to Cycloartenoid Alkaloids from Buxus Sempervirens

In a recent study aimed at compounds with antiplasmodial activity in European Box (*Buxus sempervirens* L., Buxaceae). We isolated the aminocycloartane-type alkaloid *O*-tigloylcyclovirobuxein B (**18**) which showed considerable in vitro activity against *Plasmodium falciparum* (*Pf*; IC_50_ = 0.92 µM) and some activity, yet at a much lower level, against *Tbr* (IC_50_ = 3.7 µM) [16]. In continuation of this study, a small amount of the unesterified alcohol, cyclovirobuxein B (**19**), was also isolated from this plant and tested for antiplasmodial as well as antitrypanosomal activity [17]. Surprisingly, this alkaloid displayed a very strong activity against *Tbr* with an IC_50_ value of only 0.16 µM while the antiplasmodial activity was unchanged (0.98 µM) in comparison with the ester.

**Figure 5:**
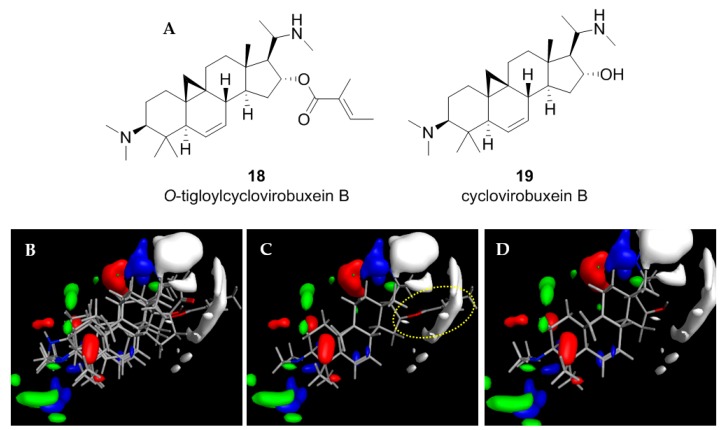
(**A**) Structures of two *Buxus sempervirens* alkaloids, **18** and **19**. (**B**) 3D-models of **18** and **19** superposed with **3**, (**C**) *O*-tigloylcyclovirobuxein B (**18**) and (**C**) cyclovirobuxein B (**19**). (**B**–**D**) show the contour maps of the CoMFA model for anti-*Tbr* activity (compare Figure 3) and the sterically unfavorable interaction of **18** explaining its low activity is marked.

In view of the structural similarity (see Figure 5A,B) between these cycloartenoid alkaloids and the steroids from *Holarrhena*, it was straightforward to assume that these compounds might address the same molecular target in *Tbr*. Molecular models of the two *Buxus*-alkaloids were therefore prepared and aligned with compound **3** in the same manner as described above. Their anti-*Tbr* activity was then predicted with the CoMFA model. The predicted pIC_50_ values (6.15 and 6.46 for **18** and **19**, respectively) were in reasonable agreement with the experimental values (5.43 and 6.79, i.e., **18** was predicted much less active than **19**) which is also reflected in their positions in the scatter plots of Figure 2). Furthermore, their interactions with the CoMFA contour maps of the QSAR model for anti-*Tbr* activity match the SAR described above very well (Figure 5B,C). In particular, the bulky tigloyl ester group of **18** explains its low antitrypanosomal activity very well since it collides with the sterically unfavorable white contour in region (c) of the model (Figure 5C) while there is no such unfavorable clash in the case of the strong trypanocide **19**, which shows similar properties to the more active *Holarrhena* alkaloids. These results thus strongly support the hypothesis that the *Buxus* alkaloids indeed affect the trypanosomes by the same mechanism of action as the *Holarrhena*-aminosteroids. Further studies aimed at isolation and activity testing of a larger variety of *Buxus* alkaloids in order to allow establishment of a refined joint 3D-QSAR have therefore been initiated.

## 3. Materials and Methods

### 3.1. Data Set

A set of 17 steroid alkaloids, all isolated from *H. africana* [1], were used for the 3D-QSAR computational studies of trypanocidal (*Tbr*) activities. The data set was divided into training (12 compounds) and test (5 compounds) sets based on random selection. Sixteen of the alkaloids, used also for 3D-QSAR of L6 cytotoxic activities, were also divided into training (12 compounds) and test (4 compounds) sets. For the 3D QSAR, the molar (M) IC_50_ values for *T. brucei rhodesiense* and L6 cytotoxicity were converted to pIC_50_ (Table 2), which is the negative decadic logarithmic value of the IC_50_ (−log_10_IC_50_) as the target (dependent variable). The structures and pIC_50_ values of all the compounds used as both training and test sets are shown in Table 1.

### 3.2. Building of Molecular Models

All 3D structures were built from fragments in the molecular operating environment, *MOE* [14]. Initial three-dimensional molecular models of all compounds were energy minimized with the MMFF94x force field and then submitted to a low-mode dynamic (LMD) conformational search using default settings of *MOE*. The resulting conformers (within an energy window of 3 kcal/mol from the global minimum) were energy minimized using the semi-empirical Austin Model 1 (AM1) Hamiltonian (MOPAC module of *MOE*) and the conformers with lowest AM1 energy were used for QSAR studies. 

### 3.3. Alignment Procedure

All structures were aligned with the lowest energy conformer of compound **3** which showed the highest antitrypanosomal (IC_50_, *Tbr* 0.075 µM) and cytotoxic (IC_50_ L6 2.48 µM) activities. The selected atoms of the steroid skeleton (carbons 3, 5, 6, 9, 13, 14, and 17, see Figure 1) were used as matching points for the superposition. All the superposed molecules and their corresponding pIC_50_ values were then converted to *Open3DQSAR*-compatible format for 3D-QSAR studies.

### 3.4. Comparative Molecular Field Analysis (CoMFA)

#### 3.4.1. Data Pretreatment

The CoMFA study was performed with the open-sourced software *Open3DQSAR* [15]. The aligned ligand assembly was automatically enclosed in a grid box exceeding the largest molecule by 5 Å in each direction and a 1 Å mesh step size was chosen for the molecular field grids. Steric (Lennard-Jones potential) and electrostatic (Coulombic potential) molecular interaction fields (MIFs) were computed with *Open3DQSAR* using MMFF94 van der Waals parameters and charges. The steric interaction field was computed using an sp^3^ carbon atom as probe while the electrostatic interaction field was computed using a volume-less probe with +1 charge. Training set MIF data were pre-filtered by setting an energy (Van der Waals and electrostatics) cutoff at ± 30 kcal/mol; variables having a standard deviation below 2.0 were discarded to minimize noise and accelerate the regression analysis [18]. Furthermore, block unscaled weighting was applied to both steric and electrostatic fields to give them the same importance in the PLS model.

#### 3.4.2. PLS Regression and Model Validation

The regression analysis of CoMFA field energies was performed using partial least squares (PLS) regression to correlate the descriptors (i.e., the MIF energies) with the pIC_50_ data by extracting five latent variables (PLS components, PCs). In order to improve the model, smart region definition was performed on the aligned molecules to reduce the dependency from grid-to-molecule reciprocal orientations. The internal validation was performed for the 5 PCs by leave-one-out (LOO) cross-validation method and the performance expressed as the coefficient of determination *Q*^2^ for the correlation between experimental and predicted pIC_50_ data of the training set.

The external predictive power of the developed CoMFA model was assessed by predicting the activities of the test set molecules, which were excluded during model development. The structural preparation of test set molecules as well as alignment and MIF calculation was the same as for the training set molecules. The activity of the test set was predicted by using the model derived from the training set where the predictive power of each PLS is expressed as the coefficient of determination *P*^2^ for the correlation between experimental and predicted pIC_50_ data of the test set. The number of significant PLS components for each model was selected on the basis of the increase in *Q*^2^ observed when adding a further constituent (see data in Table S1, Supplementary Materials). In case of the model for anti-*Tbr* activity, 3 PCs were considered significant while in case of the model for L6 cytotoxicity, 2 PCs were used.

### 3.5. Contour Mapping of Steric and Electrostatic Fields

The visualization of the results of the CoMFA model as 3D contour maps was performed using the ReadMOEGRID module of *MOE*. The contours based on the regression coefficients exported from *Open3DQSAR* were plotted with interest on where variations of steric and electrostatic properties in the structural features of the different molecules of the training set leads to the most significant increase or decrease in antitrypanosomal and cytotoxic activities. The positive and negative influences of steric interaction on activity were represented by green and white contours, respectively, while those of electrostatic interactions were denoted by blue and red contours, respectively.

### 3.6. Isolation, Characterization, and Biological Testing of Cyclovirobuxein B (**19**)

The extraction, fractionation and biological evaluation of aerial parts of *Buxus sempervirens* L. as well as the isolation, analytical and structural characterization, and bioactivity testing of *O*-tigloylcyclovirobuxein B (**18**) have been described in detail in our previous report [16]. Fraction E9 obtained by spiral coil countercurrent chromatography (spCCC) displayed promising anti-*Tbr* activity [17]. Compound **19** (cyclovirobuxein B) was detected by UHPLC/+ESI QqTOF MS as a constituent in E9 and preceding spCCC fractions (157–180 and E1–E8). It was then isolated from the pooled fractions by fast centrifugal partition chromatography (FCPC) on a Kromaton (Villejuif Cedex, France) monoaxial FCPC device with a 200 mL rotor containing 1000 metal chambers using n-hexane (upper phase): acetonitrile:dichloromethane (lower phase) 10:7:3 as solvent system in ascending mode (i.e., upper phase = mobile phase) at 1300 rotations/min and a flow rate of 2.5 mL. The separation of 55 mg of the mentioned pooled fractions led to 42 eluates (25 mL each). Compound **19** (5.3 mg) was the single constituent of eluate 23. It was characterized by UHPLC/+ESI-QqTOF-MS/MS and NMR spectroscopy (^1^H, ^13^C, COSY, HSQC, HMBC). For spectroscopic methods see [16,17]. The compound was found identical with cyclovirobuxein B which had previously been described as constituent of *B. sempervirens*, along with the tiglate **18** [19,20]. The bioassays for antitrypanosomal and antiplasmodial activity were the same as described in [1,16,17].

*Cyclovirobuxein B* (**19**) +ESI-QqTOF-MS (*m*/*z*) 415.3760 [M + H]^+^, 209.1956 [M + 2H]^2+^ (calcd. for C_27_H_47_N_2_O^+^: 415.3683; for C_27_H_48_N_2_O_2_^2+^: 208.1878); ^1^H- and ^13^C-NMR data see Supplementary Materials, Table S4. Mass- and 1D and 2D NMR spectra are shown in Figures S1–S5, Supplementary Materials.

## 4. Conclusions

The CoMFA models obtained in this study for antitrypanosomal activity against *Tbr* and for cytotoxicity of *Holarrhena* steroid alkaloids were of good statistical quality and interpretability. They explain the main structure–activity relationships within the set of investigated natural products and also provide a rationale for the considerable selectivity of several compounds against *Tbr*. Furthermore, they may allow predictions of activity and selectivity for untested steroid alkaloids in order to select further promising natural candidates and also could provide a good starting point for the rational design of (semi)synthetic analogues.

## Figures and Tables

**Table 1:** Chemical structures and activities of steroid alkaloids used for 3D quantitative structure–activity relationships (QSAR) studies.

Compounds	pIC_50_ (*Tbr*)	pIC_50_ (L6)	Compounds	pIC_50_ (*Tbr*)	pIC_50_ (L6)
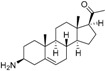 **1**	6.3958	5.2928	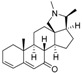 **10**	5.1326 *	4.3800 *
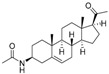 **2**	5.3159	4.8135	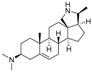 **11**	6.7781	4.2972
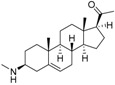 **3**	7.1249 *	5.6057 *	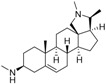 **12**	6.7781 *	4.5618
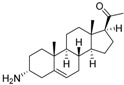 **4**	6.4320	4.7993 *	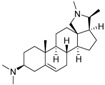 **13**	6.3778	4.2130
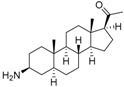 **5**	6.1726 *	4.7707	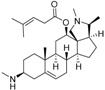 **14**	6.9245	4.8444
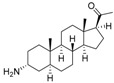 **6**	5.9190	4.7603	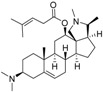 **15**	5.7807	4.0103 *
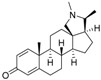 **7**	4.8282 *	n.t	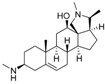 **16**	5.0856	3.7426
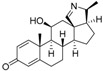 **8**	5.0624	4.1816	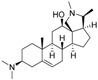 **17**	4.6798	3.9045
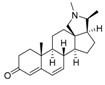 **9**	4.7568	3.8897			

* Test set compounds; pIC_50_ = −log(IC_50_) as used in the comparative molecular field analysis (CoMFA) analysis; n.t = not tested.

**Table 2:** Statistics of the CoMFA models for trypanocidal (*Tbr*) and cytotoxic (L6 cells) activities. PC is the number of latent variables (PLS components, PCs) in each model.

Model Statistics	Anti-*Tbr*, PC = 3	L6 Cytotoxic, PC = 2
*R*^2^ ± SDEC	0.995 ± 0.056	0.940 ± 0.111
*Q*^2^ ± SDEP	0.83 ± 0.33	0.64 ± 0.28
*P*^2^ ± SDEP	0.79 ± 0.51	0.59 ± 0.42
*F*-ratio	482.639 (4.066)	70.452 (4.256)
Equation of regression trendline	y = 0.621x + 2.206	y = 0.674x + 1.420

*R*^2^ = non-cross validated coefficient of determination; *Q*^2^ = coefficient of determination for leave-one-out internal cross-validation; *P*^2^ = coefficient of determination for test set predictions; *F* = Fisher value (critical F-values for the 95% probability level are reported in parentheses); SDEC = standard deviation error in calculation; SDEP = standard deviation error in prediction; PC = number of latent variables (partial least squares (PLS) components); x = actual pIC_50_; y = predicted pIC_50_.

## Supplementary Materials

The following are available online. Detailed Statistics for CoMFA models and numerical values for model predictions (Tables S1–S3), NMR data of compound **19** (Table S4) Mass and NMR spectra of compound **19** (Figures S1–S5). All molecular models and CoMFA contour maps are available from the corresponding author on request.
