# Supplementary material for: A 3D-QSAR Study on the Antitrypanosomal and Cytotoxic Activities of Steroid Alkaloids by Comparative Molecular Field Analysis
Source: Molecules. 2018 May 8;23(5):1113. doi: 10.3390/molecules23051113 (PMC6100617; doi:10.3390/molecules23051113)
Supplement: Supplementary file 1 [file molecules-23-01113-s001.pdf]

*Supplementary Material*

# A 3D-QSAR study on the antritrypanosomal and cytotoxic activities of steroid alkaloids by comparative molecular field analysis

**Charles O. Nnadi,[1,2] Julia B. Althaus,[1] Ngozi J. Nwodo,[2] Thomas J. Schmidt[1,*]**

[1] Institute of Pharmaceutical Biology and Phytochemistry (IPBP), University of Münster, PharmaCampus Corrensstraße 48, D-48149, Münster, Germany; E-mail: charles.nnadi@unn.edu.ng

[2] Department of Pharmaceutical and Medicinal Chemistry, Faculty of Pharmaceutical Sciences, University of Nigeria Nsukka, 410001 Enugu State Nigeria; E-mail: ngozi.nwodo@unn.edu.ng

* Correspondence: thomschm@uni-muenster.de; Tel.: +49-251-83-33378

**Table S1**. Statistical detaily of 3D QSAR CoMFA model for trypanocidal (*Tbr*) activity

| Model statistics/*N* | 1 | 2 | 3 | 4 | 5 |
|---|---|---|---|---|---|
| $R^2$ | 0.717 | 0.920 | **0.995** | 0.997 | 0.999 |
| SDEC | 0.405 | 0.216 | **0.056** | 0.043 | 0.028 |
| *F*-values | 25.336 | 51.473 | **482.639** | 539.039 | 917.456 |
| $Q^2$ (SDEP) | 0.42 (0.58) | 0.67 (0.44) | **0.83 (0.33)** | 0.82 (0.32) | 0.84 (0.30) |
| $P^2$ (SDEP) | 0.48 (0.67) | 0.69 (0.51) | **0.79 (0.51)** | 0.68 (0.52) | 0.69 (0.51) |

*$Q^2$ = coefficient of determination for leave-one-out cross-validation; N = number of statistical components; $R^2$ = coefficient of determination for non-cross validated model data; SDEC = standard deviation error in calculation; SDEP = standard deviation error in prediction; F = Fisher value; $P^2$ = coefficient of dermination for the test set predictions.*

**Table S2**. Statistical details of 3D QSAR CoMFA model for cytotoxic (L6) activity

| Model statistics/*N* | 1 | 2 | 3 | 4 | 5 |
|---|---|---|---|---|---|
| $R^2$ | 0.829 | **0.940** | 0.981 | 0.994 | 0.998 |
| SDEC | 0.187 | **0.111** | 0.063 | 0.034 | 0.019 |
| *F*-values | 48.562 | **70.452** | 134.159 | 302.887 | 693.318 |
| $Q^2$ (SDEP) | 0.46 (0.33) | **0.64 (0.28)** | 0.67 (0.26) | 0.70 (0.25) | 0.72 (0.24) |
| $P^2$ (SDEP) | 0.44 (0.49) | **0.59 (0.42)** | 0.74 (0.33) | 0.78 (0.30) | 0.81 (0.28) |

*$Q^2$ = coefficient of determination for leave-one-out cross-validation; N = number of statistical components; $R^2$ = coefficient of determination for non-cross validated model data; SDEC = standard deviation error in calculation; SDEP = standard deviation error in prediction; F = Fisher value; $P^2$ = coefficient of dermination for the test set predictions.*

**Table S3.** Actual vs predicted $pIC_{50}$ for anti-*Tbr* and L6 cytotoxic activity

| Compounds | $pIC_{50}$ (*Tbr*), 3PC | | $pIC_{50}$ (L6), 2PC | |
|---|---|---|---|---|
| | Actual | Predicted | Actual | Predicted |
| **1** | 6.3958 | 6.0267 | 5.2928 | 4.7213 |
| **2** | 5.3159 | 5.9046 | 4.8135 | 4.8189 |
| **3** | 7.1249* | 6.4687 | 5.6057* | 4.9596 |
| **4** | 6.4320 | 6.0267 | 4.7993* | 4.7511 |
| **5** | 6.1726* | 6.4385 | 4.7707 | 4.9136 |
| **6** | 5.9190 | 6.1137 | 4.7603 | 4.7833 |
| **7** | 4.8282* | 5.3394 | n.t | n.p |
| **8** | 5.0624 | 4.9017 | 4.1816 | 4.1339 |
| **9** | 4.7568 | 5.2974 | 3.8897 | 4.1848 |
| **10** | 5.1326* | 5.3685 | 4.3800* | 4.1369 |
| **11** | 6.7781 | 6.3490 | 4.2972 | 4.3223 |
| **12** | 6.7781* | 6.0757 | 4.5618 | 4.3017 |
| **13** | 6.3778 | 6.2482 | 4.2130 | 4.1438 |
| **14** | 6.9245 | 6.7948 | 4.8444 | 4.5317 |
| **15** | 5.7807 | 5.5249 | 4.0103* | 4.4999 |
| **16** | 5.0856 | 5.1469 | 3.7426 | 4.3146 |
| **17** | 4.6798 | 5.0961 | 3.9045 | 4.0640 |
| **18** | 5.4342* | 6.1487 | 4.7233* | 4.4447 |
| **19** | 6.7905* | 6.4609 | 4.5047* | 4.8203 |

*Test set compounds; $pIC_{50} = -\log(IC_{50})$ for CoMFA analysis; n.t = not tested; n.p = not predicted

**Table S4.** $^1$H and $^{13}$C NMR Data of cyclovirobuxein B (600 and 150 MHz, respectively, $CDCl_3$), Data are from [17].

| Position | $^1$H-NMR δ(ppm) | mult. | J (Hz) | $^{13}$C NMR δ (ppm) |
|---|---|---|---|---|
| 1 | 1,52 | *(2H) | | 31,17 |
| 2 | 1,75 | * | | 20,02 |
| | 1,53 | * | | |
| 3 | 2,06 | dd | 3,2; 11,7 | 71,40 |
| 4 | | | | 41,62 |
| 5 | 1,85 | dd | 5,49; 14,86 | 48,78 |
| 6 | 5,62 | ddd [dt] | 1,2; 1,2; 10,6 | 127,70 |
| 7 | 5,40 | ddd | 3,25; 6,1; 10,6 | 128,38 |
| 8 | 2,53 | dd | 2,0; 6,0 | 43,39 |
| 9 | | | | 20,94 |
| 10 | | | | 28,87 |
| 11 | 1,82 | dd | 5,5; 14,9 | 24,98 |
| | 1,41 | ddd | 1,57; 4,8; 14,97 | |
| 12 | 1,69 | ddd | 5; 13,4 | 32,10 |
| | 1,37 | ddd | 1,5; 5,4 13 | |
| 13 | | | | 45,56 |
| 14 | | | | 49,86 |
| 15 | 1,98 | dd | 10; 13,4 | 41,75 |
| | 1,26 | dd | 13,4; 2,1 | |
| 16 | 4,15 | ddd | 2,1; 7,8; 9,6 | 78,89 |
| 17 | 1,58 | dd | 6,7; 10,5 | 61,59 |
| 18 | 0,91 | s (3H) | | 15,71 |
| 19 | 0,72 | d | 4,1 | 18,37** |
| | -0,21 | d | 4,1 | |
| 20 | 2,49 | dq | 10,4; 6,1 | 59,15 |
| 21 | 1,09 | d(3H) | 6,1 | 18,90 |
| 28 | 0,94 | s(3H) | | 18,35** |
| 29 | 1,05 | s(3H) | | 26,17 |
| 30 | 0,79 | s(3H) | | 16,68 |
| 31/32 | 2,29 | s(6H) | | 44,31 |
| 33 | 2,44 | s(3H) | | 33,89 |

*chemical shift values extracted from HSQC spectrum due to signal overlap; **signals of C-19 and C-18 assigned with the help of an APT spectrum.

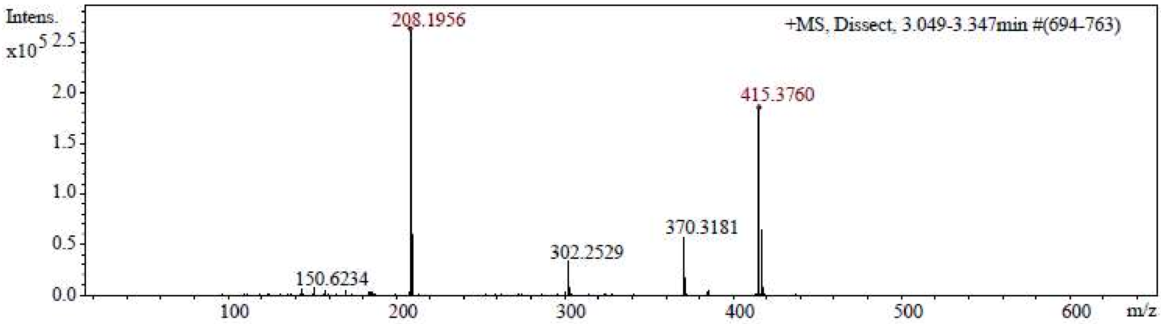


| Meas. m/z | I | z | Formula | m/z | err [mDa] | err [ppm] | mSigma | rdb | e¯ Conf |
|---|---|---|---|---|---|---|---|---|---|
| 415.3760 | 182993 | 1+ | C27 H47 N2 O | 415.3683 | -7.7 | -18.5 | 24.1 | 5.5 | even |

**Figure S1.** +ESI QqTOF mass spectrum of cylcovirobuxein B (**19**)

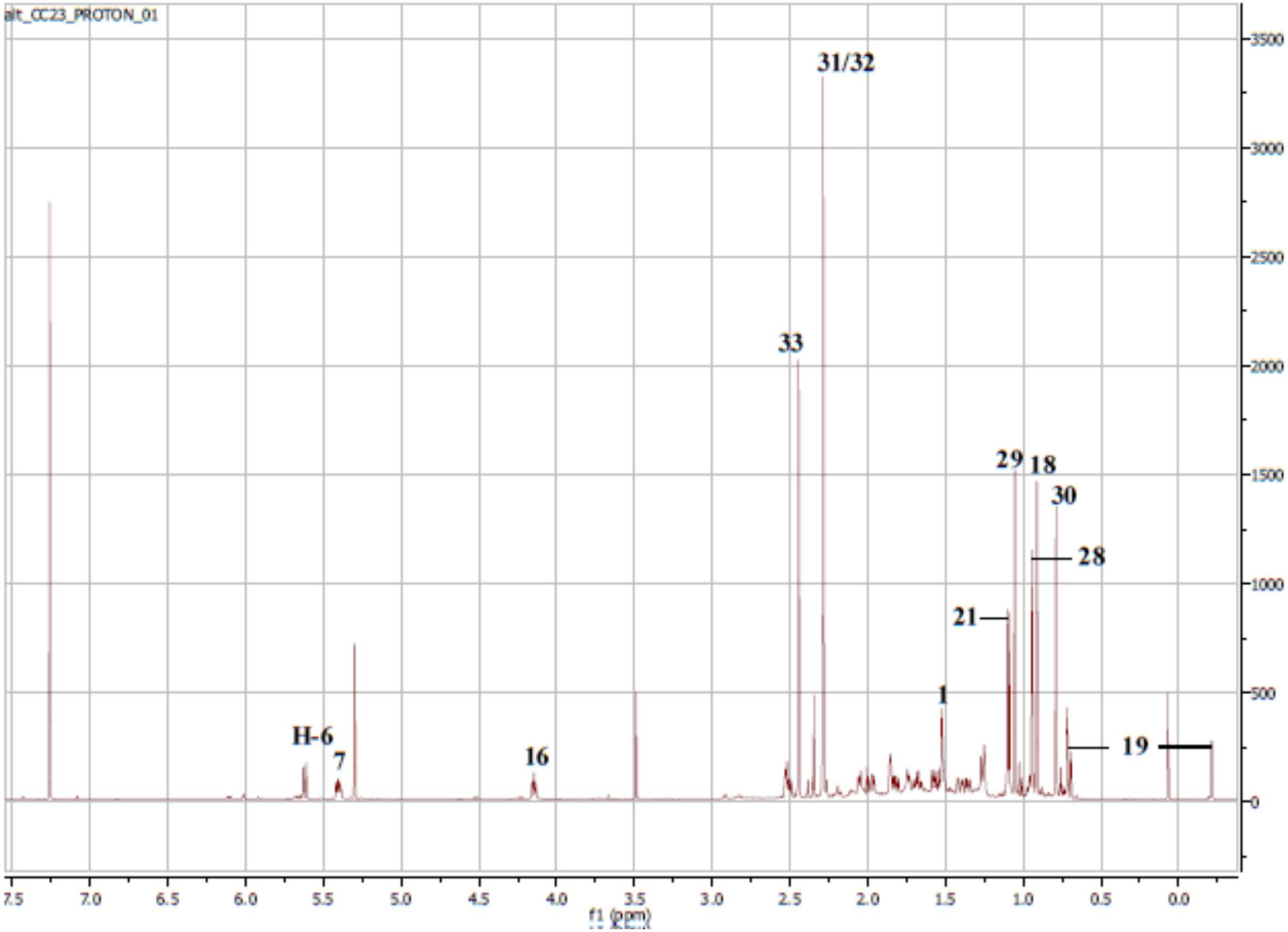


**Figure S2.** $^{1}$H NMR spectrum (600 MHz, $CDCl_3$) of cylcovirobuxein B (**19**)

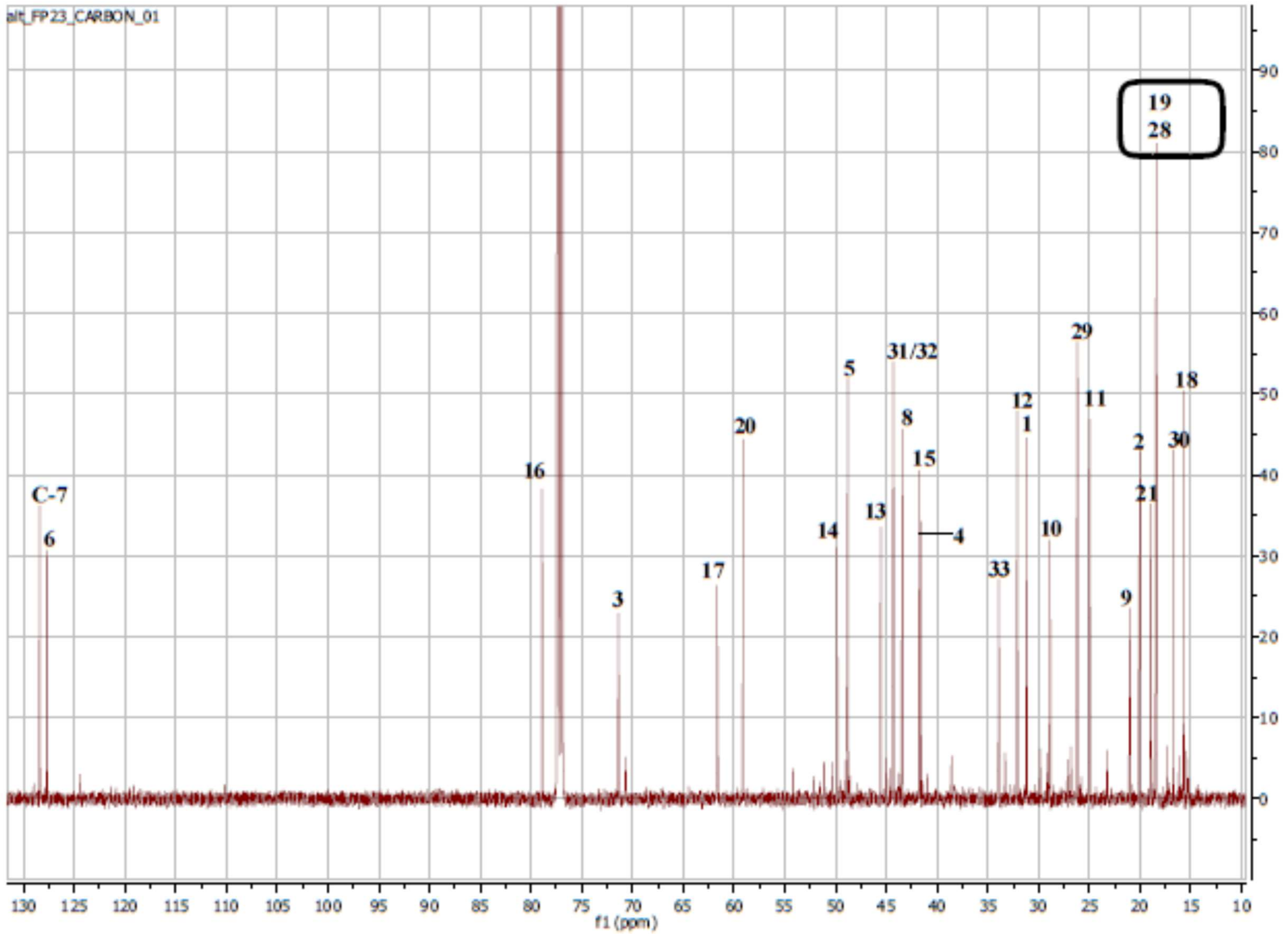


**Figure S3.** $^{13}C$ NMR spectrum (150 MHz, $CDCl_3$) of cylcovirobuxein B (**19**)

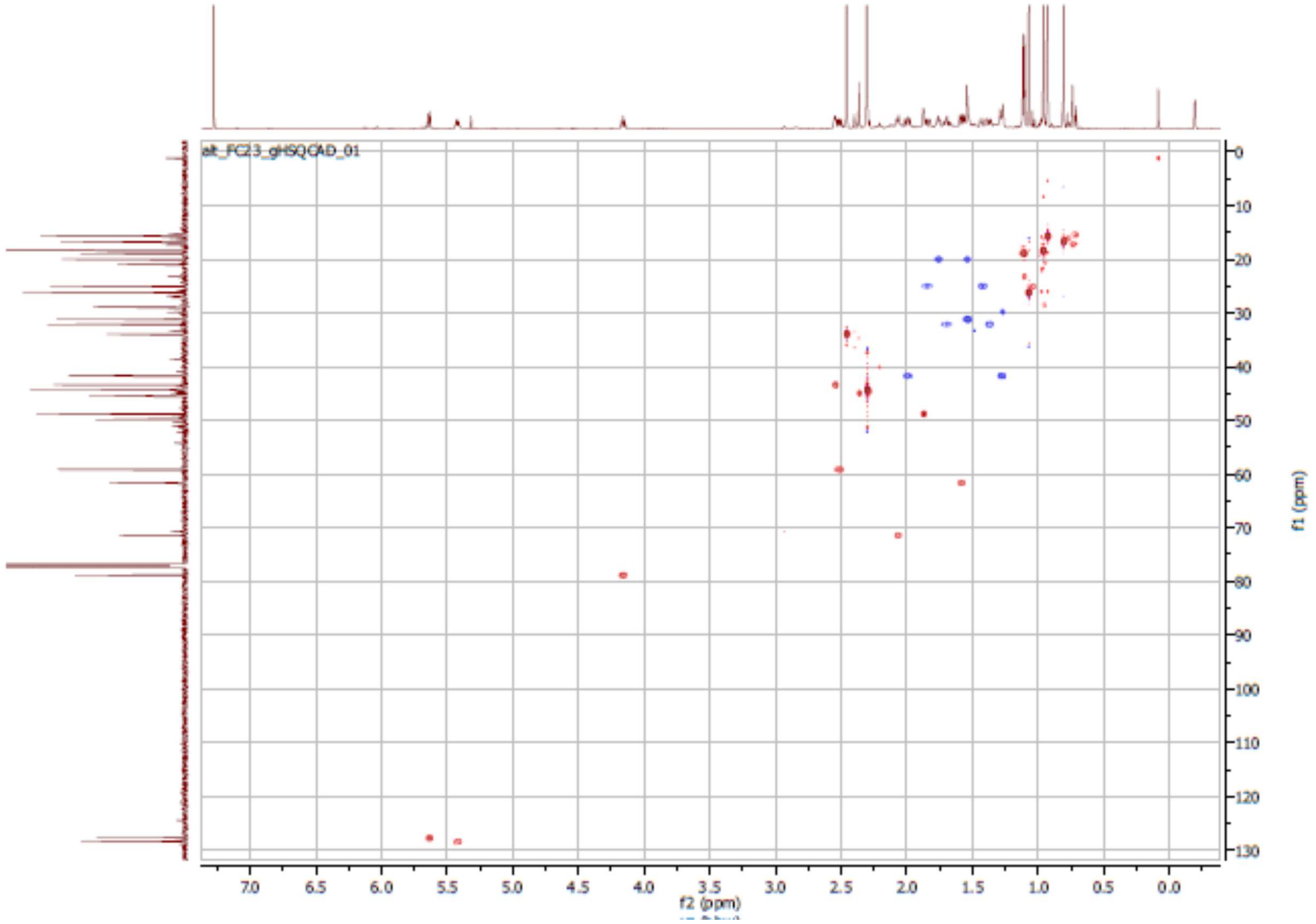


**Figure S4.** $^{1}H/^{13}C$ HSQC spectrum (600 MHz, $CDCl_3$) of cylcovirobuxein B (**19**)

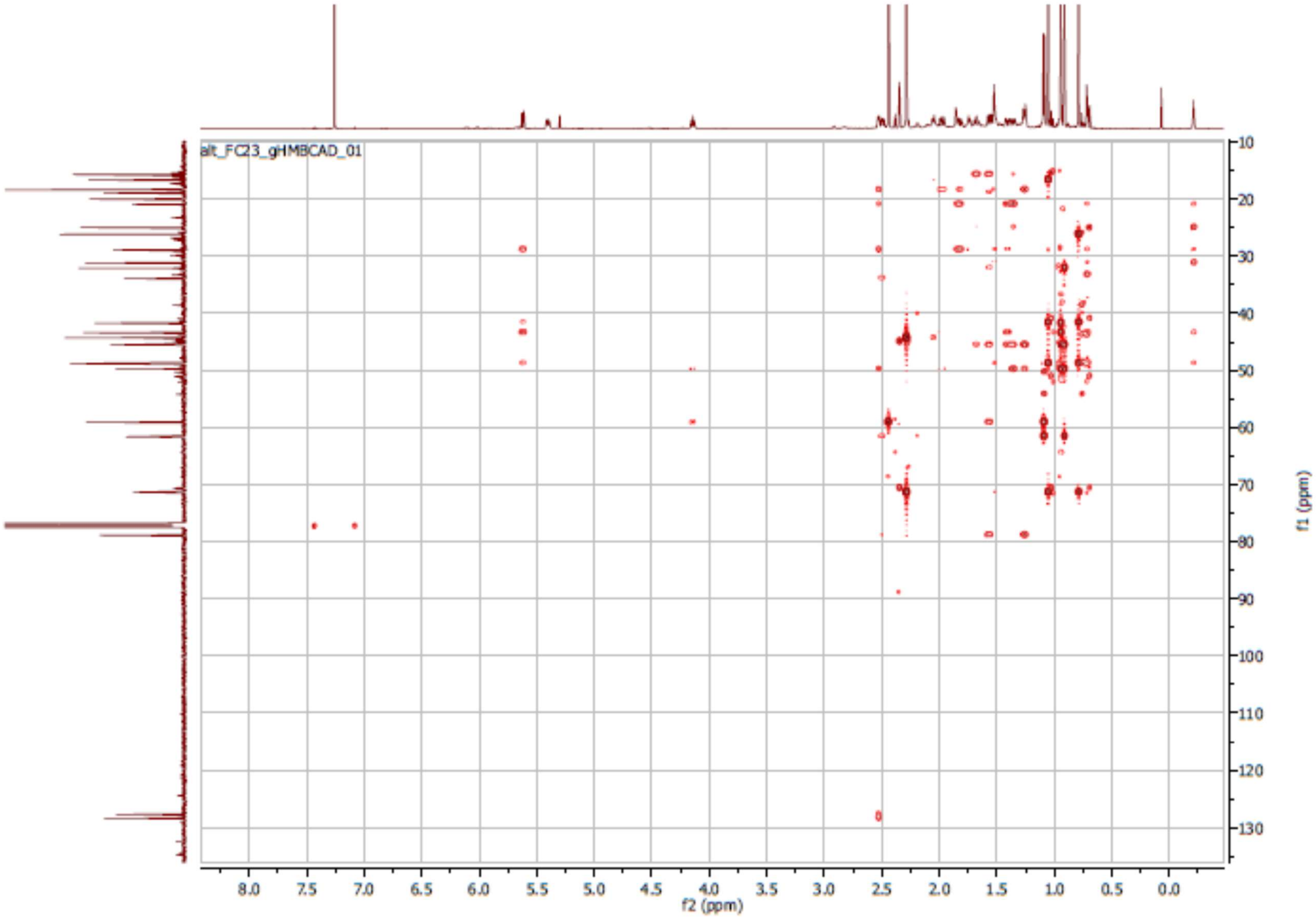


**Figure S5.** $^{1}H/^{13}C$ HMBC spectrum (600 MHz, $CDCl_3$) of cylcovirobuxein B (**19**)
